# Upregulation of microRNA-122 by farnesoid X receptor suppresses the growth of hepatocellular carcinoma cells

**DOI:** 10.1186/s12943-015-0427-9

**Published:** 2015-08-25

**Authors:** Jialin He, Kai Zhao, Lu Zheng, Zhizhen Xu, Wei Gong, Shan Chen, Xiaodong Shen, Gang Huang, Min Gao, Yijun Zeng, Yan Zhang, Fengtian He

**Affiliations:** Department of Biochemistry and Molecular Biology, College of Basic Medical Sciences, Third Military Medical University, Chongqing, 400038 China; Department of Hepatobiliary Surgery, Xinqiao Hospital, Third Military Medical University, Chongqing, 400037 China

**Keywords:** miR-122, FXR, Hepatocellular carcinoma, Cell proliferation, Gene regulation

## Abstract

**Background:**

microRNA-122 (miR-122) is the most abundant and specific miRNA in the liver. It acts as an important tumor suppressor in hepatocellular carcinoma (HCC) through regulating its target genes, but details of its own regulation are largely unknown. Farnesoid X receptor (FXR), a transcription factor with multiple functions, plays an important role in protecting against liver carcinogenesis, but it is unclear whether the anti-HCC effect of FXR is involved in the regulation of miR-122.

**Methods:**

The levels of miR-122 and FXR in HCC tissues and cell lines were examined by quantitative real-time PCR (qRT-PCR). qRT-PCR was also used to detect the expression of miR-122 target genes at mRNA level, while Western blotting was used to analyze that of their protein products. The effect of FXR on the transcriptional activity of *miR-122* promoter was evaluated by a luciferase reporter assay. Electrophoretic mobility shift assay (EMSA) and chromatin immunoprecipitation (ChIP) assay were performed to identify the FXR binding site within *miR-122* promoter region. The cell proliferation was analyzed by a CCK-8 assay. The influence of FXR on tumor growth and miR-122 expression *in vivo* was monitored using HCC xenografts in nude mice.

**Results:**

The expression of FXR was positively correlated with that of miR-122 in HCC tissues and cell lines. Activation of FXR in HCC cells upregulated miR-122 expression and in turn downregulated the expression of miR-122 target genes including insulin-like growth factor-1 receptor and cyclin G1. FXR bound directly to the DR2 element (−338 to −325) in *miR-122* promoter region, and enhanced the promoter’s transcriptional activity. Functional experiments showed that the FXR-mediated upregulation of miR-122 suppressed the proliferation of HCC cells *in vitro* and the growth of HCC xenografts *in vivo*.

**Conclusions:**

*miR-122* is a novel target gene of FXR, and the upregulation of miR-122 by FXR represses the growth of HCC cells, suggesting that FXR may serve as a key transcriptional regulator for manipulating miR-122 expression, and the FXR/miR-122 pathway may therefore be a novel target for the treatment of HCC.

**Electronic supplementary material:**

The online version of this article (doi:10.1186/s12943-015-0427-9) contains supplementary material, which is available to authorized users.

## Background

microRNAs (miRNAs), a family of small (~22-nucleotide) endogenous noncoding RNAs [1], play important roles in many cellular processes by targeting an estimated 10–30 % of all protein-coding genes [2, 3]. miRNAs regulate target genes through pairing interactions with specific mRNAs, which can lead to degradation of target mRNAs or translation repression. Recently, growing evidence has shown that a number of miRNAs are involved in the pathogenesis of human cancers.

miR-122 is the most abundant miRNA (constituting 70 % of the total miRNA population) in the liver [4–6]. It acts as an important tumor suppressor in hepatocellular carcinoma (HCC) by targeting the genes involved in cell proliferation, differentiation, apoptosis and angiogenesis [7–10]. Previous reports have also shown that its expression is specifically reduced in primary HCC [11–13], so the upregulation of miR-122 should be beneficial in the prevention and treatment of HCC.

The farnesoid X receptor (FXR), a member of the nuclear receptor superfamily, is a transcription factor with multiple functions that is mainly expressed in the liver, intestine, kidneys and adrenal glands [14]. Ligand-activated FXR binds to the response elements of target genes either as a classical FXR/retinoid X receptor alpha (RXRα) heterodimer or as a monomer [15–17], leading to changes in their expression. FXR plays an important role in regulating bile acid synthesis, and lipid and glucose metabolism [18]. Recently, it has also been shown to provide protection against liver carcinogenesis through regulating tumor-related genes such as gankyrin, nuclear factor (NF)-κB, N-myc downstream regulated gene 2 (NDRG2), p53, and carbohydrate response element binding protein [19–23]. However, it is unclear whether the anti-HCC effect of FXR is involved in the regulation of miR-122.

In the present study, we demonstrated that the level of FXR was positively correlated with that of miR-122 in HCC tissues and cell lines. FXR upregulated the expression of miR-122 by directly binding to the directed repeat separated by two nucleotides (DR2 element) (−338 to −325) in *miR-122* promoter region, indicating that *miR-122* is a novel target gene of FXR. Functional experiments showed that FXR-mediated upregulation of miR-122 suppressed the proliferation of HCC cells *in vitro* and the growth of HCC xenografts *in vivo*. These results suggest that FXR may serve as a key transcriptional regulator for manipulating miR-122 expression, and that the FXR/miR-122 pathway may be a novel target for the treatment of HCC.

## Results

### The level of FXR is positively correlated with that of miR-122 in HCC tissues and cell lines

**Fig. 1:**
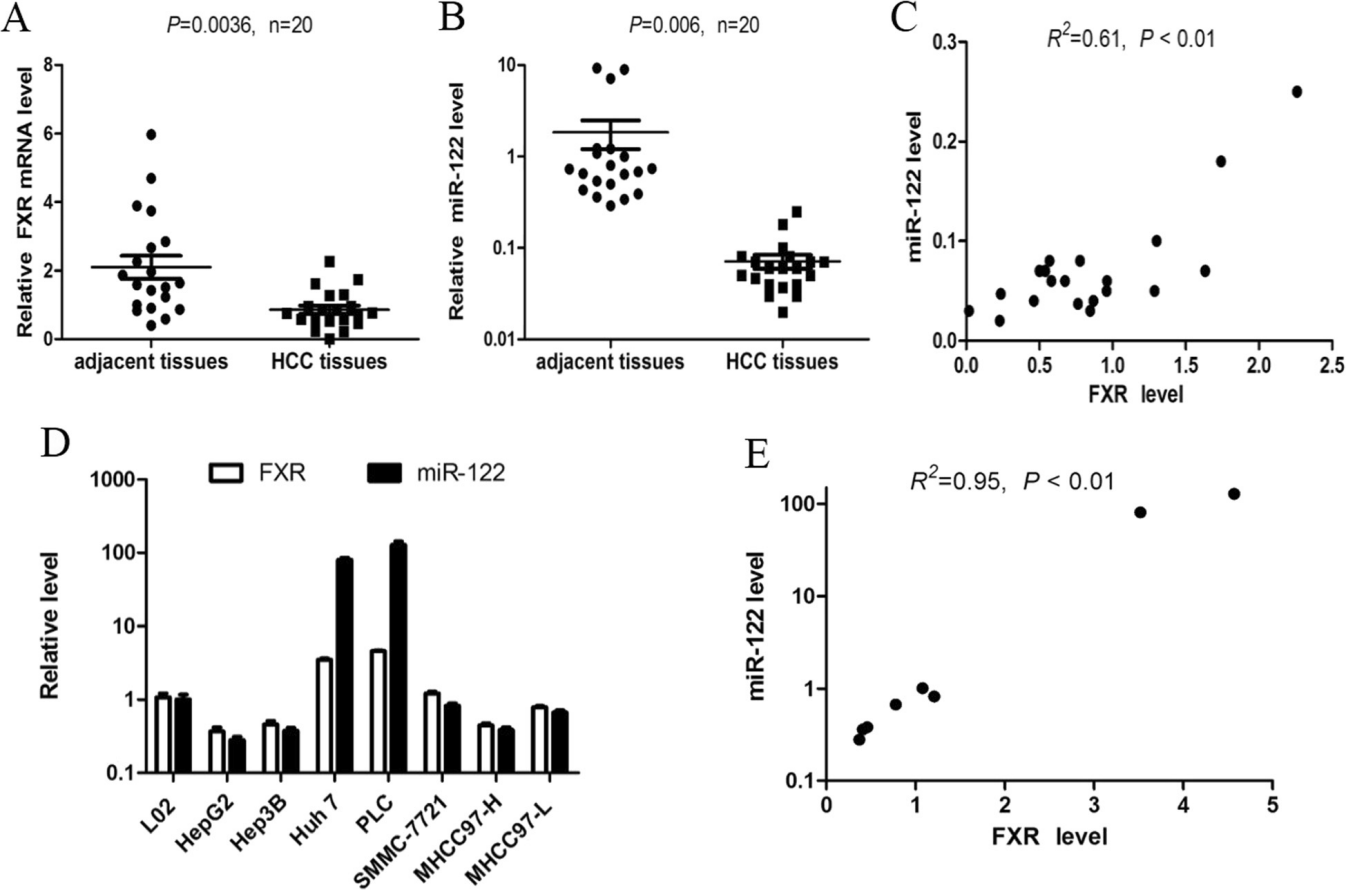
The level of FXR is positively correlated with that of miR-122. **a** and **b** The expression of FXR mRNA (**a**) and mature miR-122 (**b**) in 20 human HCC tissues and the corresponding adjacent noncancerous tissues was detected by qRT-PCR. **c** The correlation between the levels of FXR and miR-122 in HCC tissues was analyzed using Pearson’s test (*R* ^2^ = 0.61, *P* < 0.01). **d** The expression of FXR mRNA and mature miR-122 in HCC cell lines (HepG2, Hep3B, Huh7, PLC, SMMC-7721, MHCC97L and MHCC97H) and hepatic cell line L02 was assayed by qRT-PCR. **e** The correlation between the levels of FXR and miR-122 in HCC cell lines was analyzed using Pearson’s test (*R* ^2^ = 0.95, *P* < 0.01). β-actin was used as a control for FXR examination, while U6 snRNA as a control for miR-122 detection

To investigate the relationship between FXR and miR-122, their expression levels were examined using quantitative real-time PCR (qRT-PCR) in 20 human HCC tissues and the corresponding adjacent noncancerous tissues. As shown in Fig. 1a and b, the expression of both FXR and miR-122 in HCC tissues was lower than that in the adjacent noncancerous tissues. Moreover, FXR expression was positively correlated with that of miR-122 in HCC tissues (*R*^2^ = 0.61, *P* < 0.01) (Fig. 1c). Similarly, the level of FXR was also paralleled to that of miR-122 in HCC cell lines (Fig. 1d) and there was a positive correlation between them (*R*^2^ = 0.95, *P* < 0.01) (Fig. 1e). These results suggest that FXR is involved in the regulation of miR-122.

### FXR upregulates miR-122 expression and in turn downregulates the expression of miR-122 target genes in HCC cells

**Fig. 2:**
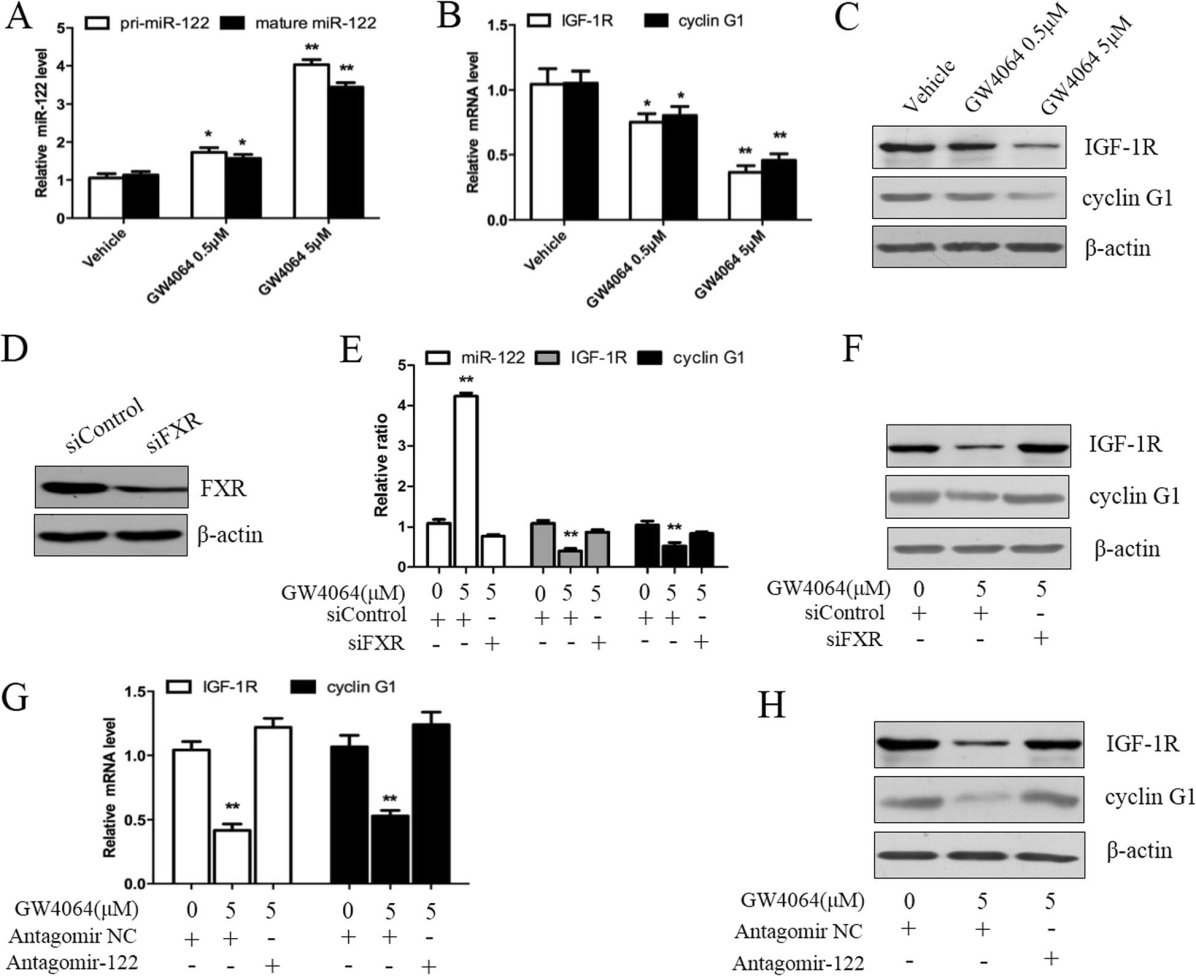
FXR upregulates miR-122 expression and suppresses the expression of miR-122 target genes. **a** Hep3B cells were treated with GW4064 (0.5 or 5 μM) or vehicle DMSO for 24 h, and then the expression of pri-miR-122 and mature miR-122 was assayed using qRT-PCR. **b** and **c** Hep3B cells were treated with GW4064 (0.5 or 5 μM) or vehicle DMSO for 48 h, and then the expression of miR-122 target genes including IGF-1R and cyclin G1 was separately examined by qRT-PCR (**b**) and Western blotting (**c**). **d** Hep3B cells were transfected with 10 nM control siRNA or FXR siRNA for 24 h, and then the level of FXR protein was detected by Western blotting. **e** and **f** After transfection with control siRNA or FXR siRNA for 24 h, Hep3B cells were treated with vehicle DMSO or 5 μM GW4064 for 24 h, and then the expression of miR-122 and its target genes including IGF-1R and cyclin G1 was determined by qRT-PCR (**e**) and Western blotting (**f**). **g** and **h** After transfection with 50 nM antagomir-122 or antagomir negative control (NC) for 6 h, Hep3B cells were treated with vehicle DMSO or 5 μM GW4064 for 48 h, and then the expression of IGF-1R and cyclin G1 was analyzed by qRT-PCR (**G**) and Western blotting (**h**). β-actin was used as a control for the examination of FXR, pri-miR-122, IGF-1R and cyclin G1, while U6 snRNA as a control for the detection of mature miR-122. *P < 0.05, **P < 0.01 *vs* vehicle

To investigate the regulation of miR-122 by FXR, Hep3B cells were treated with the FXR agonist GW4064, then the expression of miR-122 and its target genes including insulin-like growth factor-1 receptor (IGF-1R) and cyclin G1 were examined by qRT-PCR and Western blotting. As shown in Fig. 2a–c, GW4064 dose-dependently upregulated the expression of primary miR-122 (pri-miR-122) and mature miR-122 (Fig. 2a), while downregulated the expression of IGF-1R and cyclin G1 (Fig. 2b and c). Moreover, FXR activation also promoted miR-122 expression in other HCC cell lines including Huh7, HepG2, PLC and SMMC-7721 cells (Additional file 1: Figure S1). Knockdown of FXR by small interfering RNA (siRNA) significantly attenuated the GW4064-mediated upregulation of miR-122 and downregulation of its target genes (Fig. 2d–f), indicating that the effect of GW4064 on miR-122 expression is FXR-specific. Furthermore, inhibition of miR-122 by its antagomir abolished the GW4064-mediated downregulation of miR-122 target genes (Fig 2g and h), demonstrating that FXR suppressed the expression of miR-122 target genes in a miR-122-dependent manner (Namely, the FXR-induced miR-122 is functional).

### FXR enhances the transcriptional activity of *miR-122* promoter

**Fig. 3:**
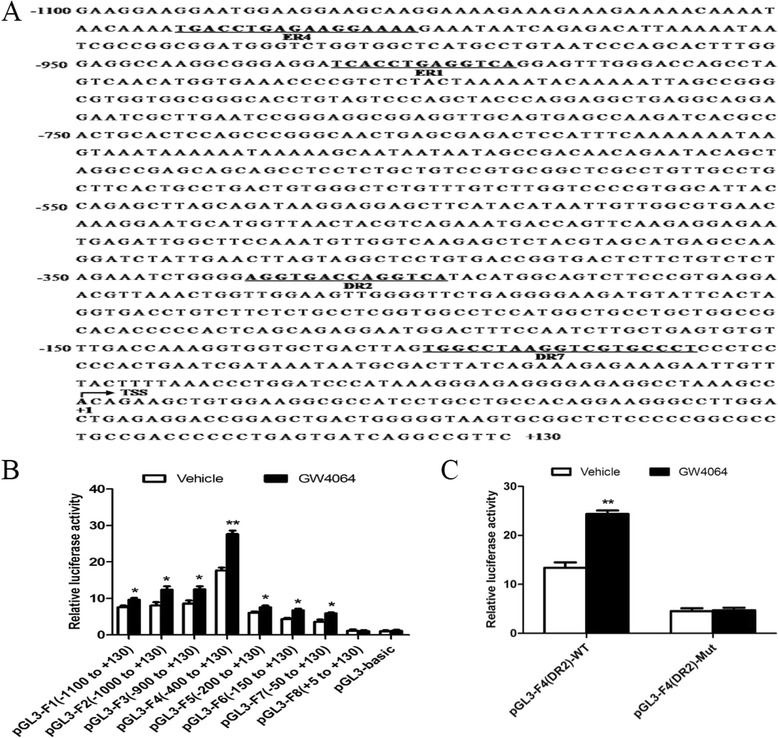
FXR enhances the transcriptional activity of *miR-122* promoter. **a** Potential FXREs in *miR-122* promoter region were predicted using an online algorithm (NUBIScan: http://www.nubiscan.unibas.ch/). Transcription start sites (TSS, +1) are indicated by an arrow. **b** Huh7 cells were co-transfected with renilla luciferase expression vector pRL-TK and one of a series of luciferase reporter constructs containing different fragments of *miR-122* promoter region. After 6 h incubation, the cells were treated with DMSO or 5 μM GW4064 for 24 h, and then dual luciferase assays were performed. Firefly luciferase activity was normalized to that of renilla luciferase. Data are shown as means ± SD from three assays performed in triplicate. **c** Huh7 cells were co-transfected with pRL-TK and either pGL3-F4(DR2)-WT (containing the wild-type DR2 element) or pGL3-F4(DR2)-Mut (containing the mutant DR2 element) for 6 h. Subsequent treatment and luciferase assays were as performed in (**b**). *P < 0.05, **P < 0.01 *vs* vehicle

To clarify whether FXR upregulates miR-122 at transcriptional level, luciferase reporter assays were performed. The putative FXR response elements (FXREs) in *miR-122* promoter region were predicted using an online algorithm (NUBIScan: http://www.nubiscan.unibas.ch/) (Fig. 3a). Based on the findings in the prediction, a series of luciferase reporters containing different fragments of the promoter region were constructed and separately transfected into HCC cells for luciferase assays. As shown in Fig. 3b, the luciferase activity of construct pGL3-F4 (containing the fragment −400 to +130) was much higher than that of the other constructs when the cells were treated with GW4064, suggesting that this region may harbor a regulatory element. Mutation of FXRE/DR2 (−338 to −325) within the construct pGL3-F4 abolished the GW4064-induced luciferase activity (Fig. 3c), indicating that the FXRE/DR2 is important for FXR-enhanced transcriptional activation of *miR-122* promoter.

### FXR binds directly to the FXRE/DR2 in *miR-122* promoter region

**Fig. 4:**
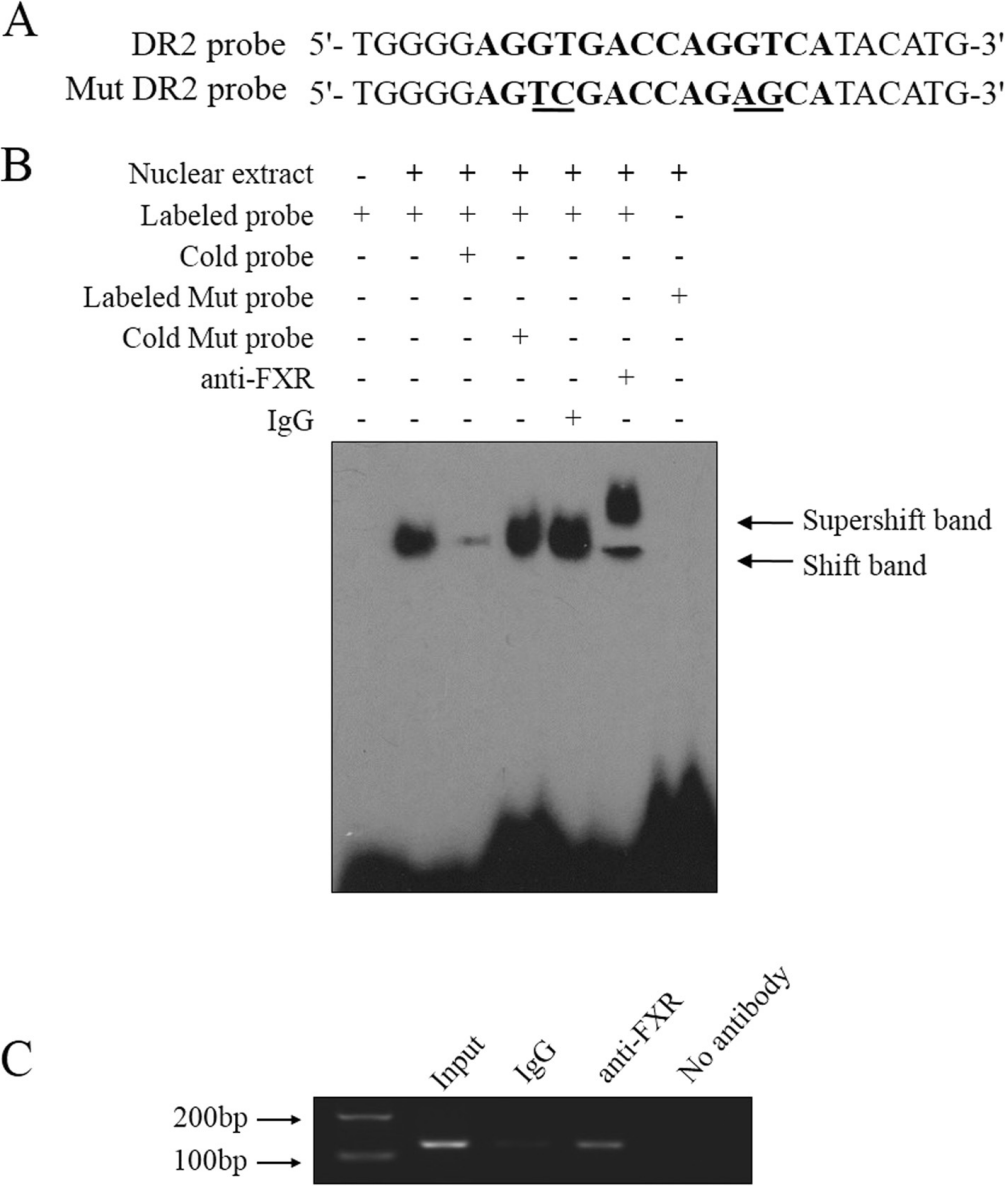
FXR binds to the FXRE/DR2 in *miR-122* promoter region. After treatment with the FXR agonist GW4064 (5 μM) for 48 h, Huh7 cells were harvested for EMSA or ChIP assays. **a** The sequences of DR2 probe and mutant (Mut) DR2 probe are shown. The DR2 element is in bold, and the mutated bases are underlined. **b** EMSA analysis of the binding of GW4064-treated Huh7 cell nuclear proteins to the FXRE/DR2. The reactions were analyzed by electrophoresis in a nondenaturing 4 % polyacrylamide gel, followed by autoradiography. The cold DR2 or Mut DR2 probe was used at 100× excess concentrations over the labeled probe in competition experiments. The antibody directed against FXR (anti-FXR) was used in supershift assays, taking IgG as a negative control. **c** ChIP assays were performed using chromatin isolated from GW4064-treated Huh7 cells. Anti-FXR was used for immunoprecipitation of the chromatin DNA fragment, taking IgG as a negative control. The precipitated DNA was extracted and amplified by PCR using primers spanning the DR2 element. The input (total DNA extract) was used as positive PCR control. No antibody (no anti-FXR and IgG in the reaction) was used as mock control

To determine the binding of FXR to the FXRE/DR2 in *miR-122* promoter region, we conducted an electrophoretic mobility shift assay (EMSA) and chromatin immunoprecipitation (ChIP) assay. The sequences of DR2 and mutant (Mut) DR2 probes were shown in Fig. 4a. EMSA revealed that the interaction of labeled DR2 probe with the nuclear extracts of HCC cells yielded a DNA/protein shift band of expected mobility (Fig. 4b). This binding was specific because it was competitively inhibited by the addition of excess unlabeled (cold) DR2 probe but not cold Mut DR2 probe, and because no obvious interaction was observed between the Mut DR2 probe and HCC cell nuclear extracts. Moreover, the addition of an anti-FXR antibody to the reaction mixture resulted in a supershift band (Fig. 4b), confirming that FXR is the protein interacting with the DR2 probe in the nuclear extracts. As shown in Fig. 4c, ChIP assay demonstrated that the anti-FXR antibody precipitated the DNA fragment containing the DR2 element, indicating that FXR binds directly to the FXRE/DR2 in *miR-122* promoter region in HCC cells.

### Inhibition of miR-122 dramatically attenuates the FXR-mediated growth suppression of HCC cells

**Fig. 5:**
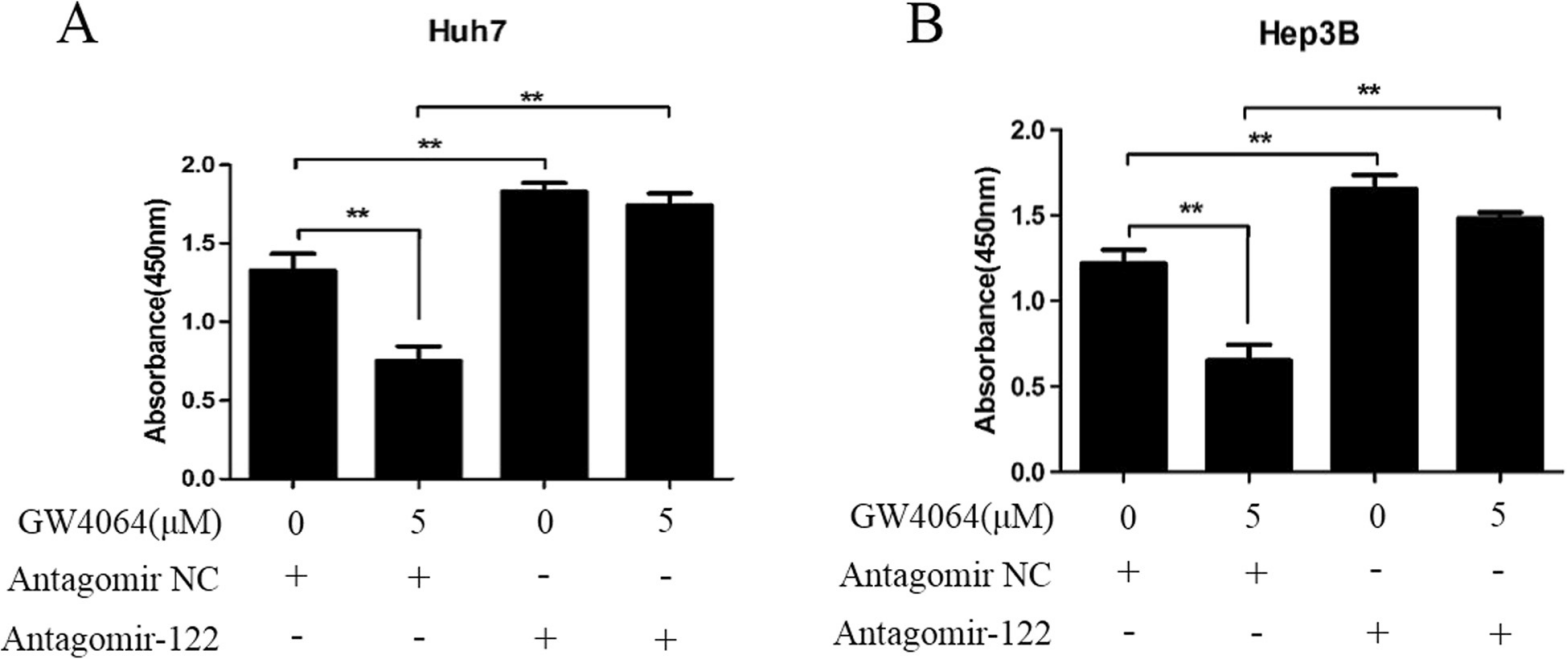
miR-122 inhibition dramatically attenuates the FXR-mediated growth suppression of HCC cells. **a** and **b** Huh7 (**a**) and Hep3B (**b**) cells were transfected with antagomir NC or antagomir-122 for 24 h, and then treated with vehicle DMSO or 5 μM GW4064 for 48 h. Subsequently, the cell proliferation was examined using the CCK-8 assay kit. Data are shown as the absorbance value at 450 nm. **P < 0.01 *vs* control

To investigate whether the repressive effect of FXR on the proliferation of HCC cells is mediated by the induction of miR-122, antagomir-122 was used in CCK-8 assays. As shown in Fig. 5, inhibition of miR-122 by its antagomir markedly attenuated the GW4064-induced growth repression of HCC cells, strongly suggesting that the FXR-mediated cell growth suppression is largely dependent on miR-122 induction.

### FXR-induced miR-122 is involved in the growth suppression of HCC xenografts *in vivo*

**Fig. 6:**
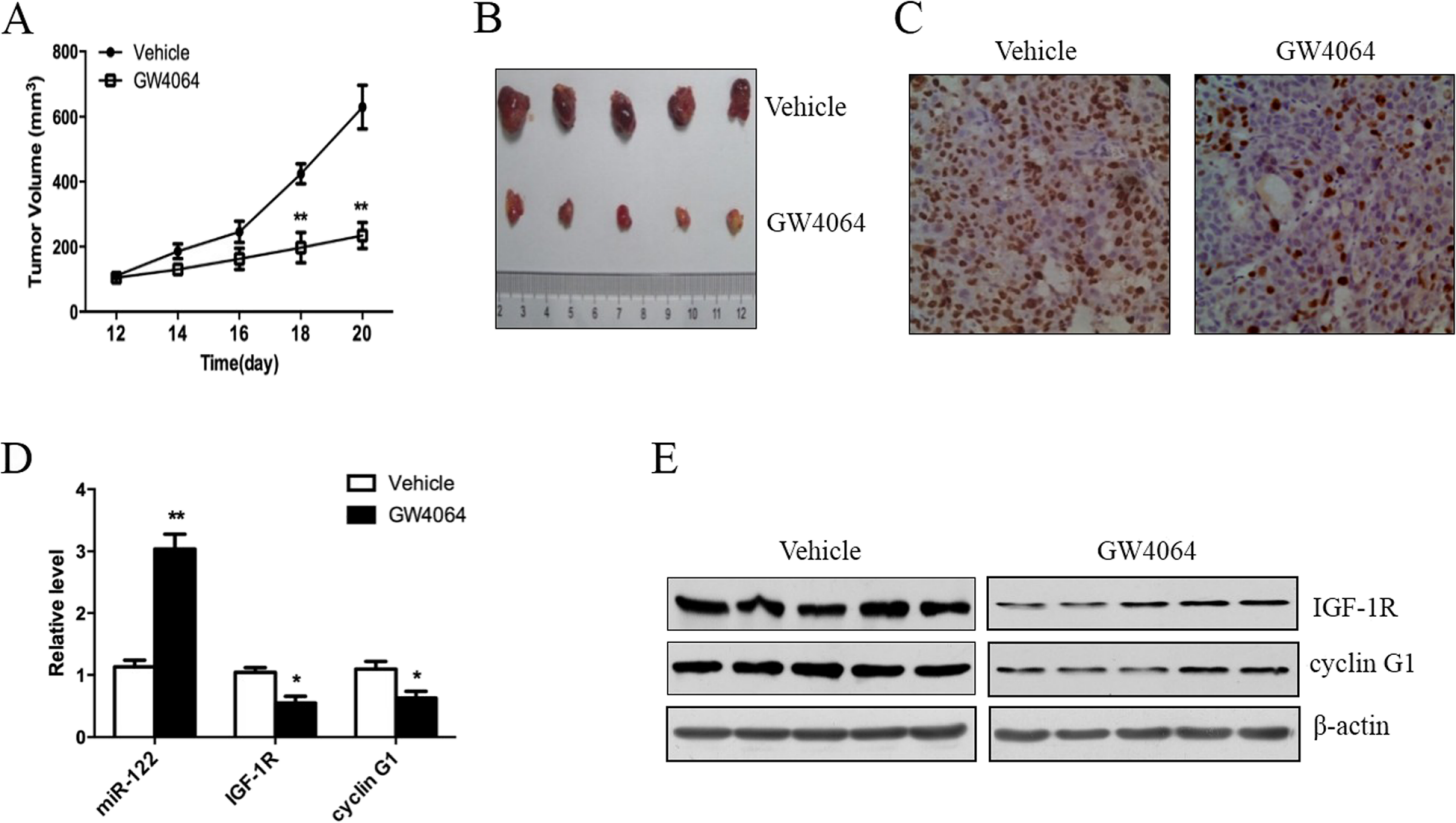
FXR agonist represses the growth of HCC xenografts and upregulates miR-122 expression *in vivo*. **a** A total of 5 × 10^6^ Hep3B cells were injected into the right axillae of each nude mouse. After 11 days, the mice were randomly assigned to different groups (*n* = 5 per group), and treated daily with vehicle or GW4064 at 40 mg/kg by intraperitoneal injection for 8 d. The xenograft tumor volume was monitored every other day. **b** HCC xenografts were harvested for size comparison. **c** The expression of Ki67 (a marker of cell proliferation) in HCC xenografts was examined by immunohistochemical staining. **d** and **e** The expression of miR-122, IGF-1R and cyclin G1 in HCC xenografts was separately detected by qRT-PCR (**d**) and Western blotting (**e**). *P < 0.05, **P < 0.01 *vs* vehicle

The above studies clearly demonstrated that the FXR-induced upregulation of miR-122 plays an important role in the FXR-mediated growth inhibition of HCC cells *in vitro*. We next investigated the effects of FXR on miR-122 induction and HCC growth *in vivo*. As shown in Fig. 6a and b, treatment with GW4064 dramatically repressed the growth of HCC xenografts in nude mice. Immunohistochemical staining showed that GW4064 significantly reduced the expression of Ki67 (a marker of proliferation) in the HCC xenografts (Fig. 6c). Consistent with the *in vitro* findings, the activation of FXR by GW4064 *in vivo* markedly increased miR-122 expression and decreased that of miR-122 target genes including IGF-1R and cyclin G1 in HCC xenografts (Fig. 6d and e). These results suggest that FXR exerts its anti-HCC effects through upregulating miR-122 expression *in vivo*.

## Discussion

miR-122 is highly enriched in the liver, and plays an important role in regulating hepatocyte development, differentiation, lipid metabolism, and stress responses [24–27]. Aberrant expression of miR-122 is closely related with liver diseases. For examples, miR-122 represses hepatitis B virus (HBV) replication, and is decreased in the livers of HBV-positive patients [28, 29]. Moreover, miR-122 is an important tumor suppressor of HCC [11, 13], and its downregulation in human HCC tissues [8, 12] is associated with metastasis and poor prognosis [30, 31]. Because of many important roles of miR-122, it is necessary to understand its regulatory mechanisms. Previous studies have shown that the transcriptional factors hepatocyte nuclear factor 4 alpha (HNF4α) and CCAAT/enhancer binding protein alpha (C/EBPα) can modulate miR-122 expression [30, 32].

The nuclear receptor FXR, a multiple functional transcription factor, has been received increasing attention as a therapeutic target for the treatment of liver carcinoma and other disorders such as metabolic diseases and fibrosis. In this study, we show for the first time that FXR upregulates miR-122 expression by binding directly to the DR2 element in *miR-122* promoter region, and, moreover, that this upregulation plays an important role in the FXR-mediated growth suppression of HCC cells *in vitro* and *in vivo*.

Recently, Song et al. demonstrated that the nuclear receptor peroxisome proliferator-activated receptor gamma (PPARγ) epigenetically regulates miR-122 expression in HCC cells [33]. They found that treatment with the DNA methylation inhibitor 5′-aza-2′deoxycytidine and histone deacetylation inhibitor 4-phenylbutyric acid increased the association of PPARγ/RXRα, but decreased that of its corepressors (N-CoR and SMRT), with miR-122 regulatory elements, leading to an upregulation of miR-122 transcription. FXR and PPARγ share a number of characteristics, including acting as a heterodimer with RXRα, although FXR can also bind to the response elements as a monomer, and recruits different cofactors from those of PPARγ to regulate target genes. Therefore, it is not clear whether FXR can epigenetically regulate miR-122 expression in the same way as PPARγ, which requires further study.

Besides its regulation on miR-122, FXR uses other mechanisms to protect against HCC including the repression of NF-κB activation in hepatocytes [20], indicating that its anti-inflammatory properties may contribute to HCC prevention. Moreover, FXR also inhibits the expression of gankyrin, a small proteasome subunit that mediates the downregulation of tumor suppressor proteins such as Rb, p53, HNF4α and C/EBPα in the development of HCC [34]. Additionally, FXR directly promotes the expression of HCC suppressors such as small heterodimer partner and NDRG2, leading to the repression of HCC development, growth and metastasis [35–37, 21]. These reports highlight the complexity of FXR anti-HCC mechanisms, which should be investigated further.

Many target genes of miR-122 are involved in hepatocarcinogenesis, HCC growth and metastasis, including IGF-1R, cyclin G1, Wnt1, serum response factor, a disintegrin and metalloprotease 10 (ADAM10), ADAM17, cut-like homeobox 1, pyruvate kinase muscle isozyme 2, and pituitary tumor-transforming gene 1 binding factor [24, 38, 39]. Of these, cyclin G1 and IGF-1R are involved in cell proliferation. Cyclin G1 negatively regulates p53 protein by recruiting B subunit of phosphatase 2A to dephosphorylate Mdm-2 [40]. Overexpression of cyclin G1 enhances the growth of cancer cells, while its silencing suppresses cell proliferation [41]. It can be directly downregulated by miR-122, and the expression of cyclin G1 and miR-122 is inversely correlated in HCC tissues [42]. IGF-1R has recently been proposed as a novel target for cancer treatment because it is overexpressed in a range of cancers [43, 44]. miR-122 suppresses IGF-1R expression and attenuates IGF-1R/Akt signaling, which sustains the activity of glycogen synthase kinase 3 beta and in turn represses cancer cell proliferation [30].

## Conclusions

Our data show that the level of FXR is positively correlated with that of miR-122 in HCC tissues and cell lines. FXR upregulates the expression of miR-122 in HCC cells by binding directly to the DR2 element (−338 to −325) in *miR-122* promoter region, which in turn downregulates the expression of miR-122 target genes including IGF-1R and cyclin G1. The FXR-mediated upregulation of miR-122 suppresses the proliferation of HCC cells *in vitro* and the growth of HCC xenografts *in vivo* in nude mice. Although more studies are warranted to understand the detailed molecular mechanisms by which miR-122 regulates its target genes in HCC cells, our findings demonstrate that miR-122 is a novel target gene of FXR. These results also suggest that FXR could serve as a key transcriptional regulator for manipulating miR-122 expression, and that the FXR/miR-122 pathway may be a novel target for the treatment of HCC.

## Materials and methods

### Reagents

The FXR agonist GW4064 was purchased from Sigma Chemical Company (St Louis, MO). Antagomir-122 (5′-CAAACACCAUUGUCACACUCCA-3′), antagomir negative control (5′-CAGUACUUUUGUGUAGUACAA-3′), siRNA for FXR (5′-CCUCAGGAAAUAACAAAUATT-3′), and siRNA negative control (5′-UUCUCCGAACGUGUCACGUTT-3′) were synthesized by GenePharma (Shanghai, China). The all-in-one miRNA quantitative reverse transcriptase PCR detection kit was purchased from GeneCopoeia (Guangzhou, China). The dual luciferase assay system was from Promega (Madison, WI). The ChIP kit was from Millipore (Billerica, MA). Antibodies against FXR (sc-1204), IGF-1R (sc-712), cyclin G1 (sc-7865) and β-actin (sc-47778) were purchased from Santa Cruz Biotechnology (Dallas, TX).

### Patient tissues and cell lines

A total of 20 HCC tissues and the corresponding adjacent noncancerous tissues were obtained from Department of Hepatobiliary Surgery, Xinqiao Hospital, Third Military Medical University (Chongqing, China). Fresh tissue samples were collected and snap frozen in liquid nitrogen. The study was approved by the ethics committee of Third Military Medical University (Chongqing, China).

Human HCC cell lines HepG2 and Hep3B were from American Type Culture Collection (Manassas, VA). The other HCC cell lines including Huh7, PLC, SMMC-7721, MHCC97L and MHCC97H, and hepatic cell line L02 were purchased from China Center for Type Culture Collection (Wuhan, China). All cells were cultured in Dulbecco’s Modified Eagle’s medium supplemented with 10 % fetal bovine serum, streptomycin (100 mg/mL) and penicillin (100 U/mL) at 37 °C in a 5 % CO_2_ humid incubator.

### Quantitative real-time PCR (qRT-PCR)

The expression of mature miR-122 was assayed using an all-in-one miRNA quantitative reverse transcriptase PCR detection kit according to the manufacturer’s protocol, normalizing to U6 small nuclear RNA (snRNA). For examination of the mRNAs (including FXR, IGF-1R and cyclin G1 mRNAs) and pri-miR-122, total RNA was extracted with TRIzol reagent (Invitrogen, Carlsbad, CA), and then the first-strand cDNA was synthesized using M-MLV reverse transcriptase (Invitrogen). Real-time PCR was performed with SYBR green qPCR master mix (Promega). Relative levels of the mRNAs and pri-miR-122 were normalized to that of β-actin mRNA. The primer sets for qRT-PCR are listed in Additional file 1: Table S1.

### Western blotting

Whole proteins were extracted from cells or tissues, and protein concentrations were determined using the Bradford protein assay kit (Beyotime, Shanghai, China). The proteins (50 μg/lane) were separated by 10 % sodium dodecyl sulfate (SDS) polyacrylamide gel electrophoresis (PAGE) and transferred to polyvinylidene difluoride membranes (Millipore). Subsequently, the membranes were blocked with 5 % fat-free dry milk in Tris-buffered saline containing 0.1 % Tween-20, and then incubated separately with primary antibodies against FXR, IGF-1R, cyclin G1 or β-actin at 4 °C overnight. After incubation with horseradish peroxidase-conjugated secondary antibodies for 1 h, enhanced chemiluminescence detection reagents (Pierce, Rockford, IL) were used to visualize the signals.

### Plasmid construction and luciferase reporter assay

Putative FXREs in human *miR-122* promoter region were predicted using an online algorithm (NUBIScan: http://www.nubiscan.unibas.ch/). Based on this prediction (Fig. 3a), different lengths of human *miR-122* promoter region were amplified by PCR using Huh7 cell genomic DNA as a template (The primer sequences are listed in Additional file 1: Table S2). The fragments were then separately inserted between *Kpn*I and *Hin*dIII sites of the pGL3-basic vector (Promega), and the resulting plasmids were named as follows with the fragment of *miR-122* promoter region specified: pGL3-F1 (−1100 to +130), pGL3-F2 (−1000 to +130), pGL3-F3 (−900 to +130), pGL3-F4 (−400 to +130) (also named pGL3-F4(DR2)-WT), pGL3-F5 (−200 to +130), pGL3-F6 (−150 to +130), pGL3-F7 (−50 to +130) and pGL3-F8 (+5 to +130). pGL3-F4(DR2)-Mut, derived from pGL3-F4(DR2)-WT, contained mutations in the DR2 element (AATCGACCAGACTA, the mutated bases are underlined).

For luciferase reporter assays, the above plasmids were separately co-transfected with the renilla luciferase expression vector pRL-TK (Promega) into Huh7 cells using Lipofectamine 2000 (Invitrogen) according to the manufacturer’s protocol. After 6 h incubation, the cells were treated with vehicle dimethyl sulfoxide (DMSO) or GW4064 (5 μM) for 24 h. The cells were then harvested for the detection of luciferase activity using the dual-luciferase assay kit (Promega) according to the manufacturer’s instructions. Firefly luciferase activity was normalized to that of renilla luciferase activity. All transfection experiments were performed in triplicate and repeated at least three times.

### EMSA assay

Nuclear extracts were prepared from GW4064-treated Huh7 cells using the NE-PER nuclear and cytoplasmic extraction kit (Pierce), and the protein concentrations were determined using Bradford protein assay kit (Beyotime). The double-stranded probes were end-labeled with [γ-^32^P]-ATP using T4 polynucleotide kinase (Takara, Shiga, Japan). The binding reactions were performed separately in a 15 μl reaction mixture containing 5× gel shift binding buffer (5 mM MgCl_2_, 2.5 mM EDTA, 2.5 mM DTT, 250 mM NaCl, 50 mM Tris–HCl (pH7.5), 25 mg/ml poly(dI-dC) and 20 % glycerol (v/v)), and 5 μg nuclear proteins. For competition experiments, unlabeled (cold) DR2 or Mut DR2 probe was added to the reaction mixture at 100× excess concentrations over the labeled probe. The mixtures were then incubated at room temperature for 10 min. For supershift assays, 4 μg antibody against FXR or control IgG was added to the reaction mixture and incubated on ice for 30 min. Subsequently, 6000 cpm of ^32^P-labeled probe was added to each reaction mixture and incubated at room temperature for 20 min. All the reaction products were analyzed by electrophoresis in a 4 % nondenaturing polyacrylamide gel (59 : 1, acrylamide : bisacrylamide) in 0.5× Tris-borate-EDTA. The gel was then dried and exposed to x-ray film overnight at −70 °C for autoradiography.

### ChIP assay

ChIP assays were performed using the ChIP Assay kit according to the manufacturer’s instructions. Briefly, Huh7 cells were treated with 5 μM GW4064 for 48 h, and then incubated with formaldehyde at a final concentration of 1 % (v/v) for 10 min at 37 °C to cross-link the nuclear proteins to DNA. Subsequently, the cells were harvested by centrifugation at 4 °C for 4 min at 1000 × g, and then lysed in 200 μl SDS lysis buffer (1 % SDS, 10 mM EDTA and 50 mM Tris–HCl (pH 8.1)). Chromation sonication was performed to shear the DNA to an average length of 200–1000 bp, followed by the immunoprecipitation with the antibody against FXR, taking IgG as a control. The precipitated DNA was extracted and subjected to PCR amplification using the primer pair spanning the FXRE/DR2 in *miR-122* promoter region (−390 to −261) (forward primer: 5′-AACTTAGTAGGCTCCTGTGACCGG − 3′, and reverse primer: 5′ − ATCTTCCCCTCAGAAC CCCAACT − 3′).

### Cell proliferation assay

The cell proliferation was examined using the CCK-8 assay kit (Beyotime) according to the manufacturer’s instructions. Briefly, Huh7 or Hep3B cells were seeded onto 96-well plates (2 × 10^3^ cells/well) and transfected with antagomir-122 or the control antagomir (NC) for 24 h. The cells were then treated with vehicle DMSO or 5 μM GW4064 for 48 h, followed by the addition of 10 μL WST-8 dye to each well. After incubation at 37 °C for 4 h, the absorbance value at 450 nm was determined using a microplate reader.

### *In vivo* experiments

Eight-week-old male nude mice were purchased from the Laboratory Animal Center of China (Shanghai, China) and cared for under the guidelines of the Animal Care Committee of Third Military Medical University (Chongqing, China). A total of 5 × 10^6^ Hep3B cells in 150 μl phosphate-buffered saline were subcutaneously injected into the right axilla of each nude mouse. After 11 days, the mice were randomly assigned to control and test groups (*n* = 5 per group). They then received a daily intraperitoneal injection of 40 mg/kg GW4064 (test group) or vehicle (control group) for 8 d. The length and width of the xenograft tumors were monitored every other day, and their volumes were estimated using the following formula: volume = width^2^ × length × 1/2. Subsequently, the mice were sacrificed, and the tumors were harvested for analysis of the expression of Ki67, miR-122 and its target genes including IGF-1R and cyclin G1.

### Immunohistochemical staining

The harvested xenograft tumors were fixed with 4 % polyoxymethylene, and then paraffin-embedded and sectioned. The sections were incubated with the primary antibody against Ki67 (ZSGB-BIO, Beijing, China), followed by a peroxidase-conjugated secondary antibody. 3,3′-diaminobenzidine was used to visualize the Ki67 signal, and the sections were observed under Olympus IX81 photomicroscope.

### Statistical analysis

All data were expressed as means ± SD unless otherwise stated. Statistical analysis was performed using SPSS13.0 software. Differences between two groups were determined by the Student’s *t*-test, and correlation analysis was performed using Pearson’s test. *P* < 0.05 was defined as statistically significant.

## Additional file

Additional file 1: Figure S1. Activation of FXR upregulates miR-122 expression in HCC cells. HepG2, Huh7, PLC and SMMC-7721 cells were separately treated with GW4064 (5 μM) or vehicle DMSO for 24 h, and then the expression of pri-miR-122 (A) and mature miR-122 (B) was examined by qRT-PCR. ***P* < 0.01 *vs* vehicle. Table S1. The primer sets for qRT-PCR. Table S2. The primer sets for PCR amplification of different fragments of human *miR-122* promoter region. (DOC 82 kb)

## Abbreviations

HCC: Hepatocellular carcinoma
FXR: Farnesoid X receptor
miR-122: microRNA-122
pri-miR-122: primary miR-122
FXRE: FXR response element
DR2: Directed repeat separated by two nucleotides
IGF-1R: Insulin-like growth factor-1 receptor
EMSA: Electrophoretic mobility shift assay
ChIP: Chromatin immunoprecipitation
RXRα: Retinoid X receptor alpha
NDRG2: N-myc downstream-regulated gene 2
HNF4α: Hepatocyte nuclear factor 4 alpha
C/EBPα: CCAAT/enhancer binding protein alpha
PPARγ: Peroxisome proliferator-activated receptor gamma
N-CoR: Nuclear receptor corepressor
SMRT: Silencing mediator of retinoid and thyroid receptor
ADAM: A disintegrin and metalloprotease
Mdm-2: Murine double minute 2
snRNA: small nuclear RNA
