# Supplementary material for: Upregulation of microRNA-122 by farnesoid X receptor suppresses the growth of hepatocellular carcinoma cells
Source: Mol Cancer. 2015 Aug 25;14:163. doi: 10.1186/s12943-015-0427-9 (PMC4547435; doi:10.1186/s12943-015-0427-9)
Supplement: Additional file 1: Figure S1. — Activation of FXR upregulates miR-122 expression in HCC cells. HepG2, Huh7, PLC and SMMC-7721 cells were separately treated with GW4064 (5 μM) or vehicle DMSO for 24 h, and then the expression of pri-miR-122 (A) and mature miR-122 (B) was examined by qRT-PCR. **P < 0.01 vs vehicle. Table S1. The primer sets for qRT-PCR. Table S2. The primer sets for PCR amplification of different fragments of human miR-122 promoter region. (DOC 82 kb) [file 12943_2015_427_MOESM1_ESM.doc]

Additional file 1

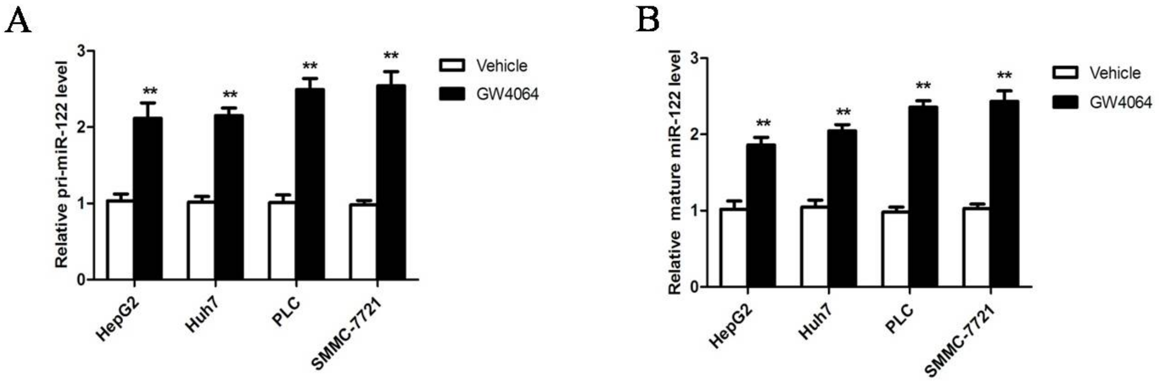

Figure S1. Activation of FXR upregulates miR-122 expression in HCC cells. HepG2, Huh7, PLC and SMMC-7721 cells were separately treated with GW4064 (5 μM) or vehicle DMSO for 24 h, and then the expression of pri-miR-122 **(A)** and mature miR-122 **(B)** was examined by qRT-PCR. $^{**}P < 0.01$ *vs* vehicle.

Table S1. The primer sets for qRT-PCR

Gene (human)	Forward primer	Reverse primer
FXR	5′- GCGCGTCAGCAGGGAGGATC-3′	5′-TCCATTCATTCGGCCAGATTAT-3′
mature miR-122	5′-TGGAGTGTGACAATGGTGTTTG-3′	Universal Adaptor PCR Primer (supplied in the kit)
pri-miR-122	5′-AATGGTGGAATGTGGAGGTGAAG-3′	5′-AGGGAAGGATTGCCTAGCAGTAG-3′
IGF-1R	5′- TGGCATACCTCAACGCCAAT-3′	5′- AGGACCAGACGTCCGAGTAA-3′
Cyclin G1	5′- TGGCATACCTCAACGCCAAT-3′	5′- AGGACCAGACGTCCGAGTAA-3′
β-actin	5′- GTGAAGGTGACAGCAGTCGGTT-3′	5′- GAAGTGGGGTGGCTTTTAGGA-3′
U6 snRNA	5′-CGCTTCGGCAGCACATATACTAA-3′	5′-TATGGAACGCTTCACGAATTTGC-3′

Table S2. The primer sets for PCR amplification of different fragments of human *miR-122* promoter region

Fragment	Forward primer	Reverse primer
F1(-1100 to +130)	5′-CGGGGTACCGAAGGAAGGAATGGAAGGAAGC-3′	5′-CCCAAGCTTGAACGGCCTGATCACTCAGG-3′
F2(-1000 to +130)	5′-CGGGGTACCTCTGGGTAGGCGGCCGCT-3′	5′-CCCAAGCTTGAACGGCCTGATCACTCAGG-3′
F3(-900 to +130)	5′-CGGGGTACCCTGCCCCAAAGTGGTACAACTG-3′	5′-CCCAAGCTTGAACGGCCTGATCACTCAGG-3′
F4(-400 to +130)	5′-CGGGGTACCGGATCTATTGAACTTAGTAGGC-3′	5′-CCCAAGCTTGAACGGCCTGATCACTCAGG-3′
F5(-200 to +130)	5′-CGGGGTACCCACACCCCCACTCAGCAGAG-3′	5′-CCCAAGCTTGAACGGCCTGATCACTCAGG-3′
F6(-150 to +130)	5′-CGGGGTACCTTGACCAAAGGTGGTGCTGAC-3′	5′-CCCAAGCTTGAACGGCCTGATCACTCAGG-3′
F7(-50 to +130)	5′-CGGGGTACCTACTTTTAAACCCTGGATCCCA-3′	5′-CCCAAGCTTGAACGGCCTGATCACTCAGG-3′
F8(+5 to +130)	5′-CGGGGTACCGCTTCGGCAGCACATATACTAA-3′	5′-CCCAAGCTTGAACGGCCTGATCACTCAGG-3′
